# The Actin-Capping Protein Alpha-Adducin Is Required for T-Cell Costimulation

**DOI:** 10.3389/fimmu.2019.02706

**Published:** 2019-11-20

**Authors:** Timothy J. Thauland, Humza A. Khan, Manish J. Butte

**Affiliations:** Division of Immunology, Allergy, and Rheumatology, Department of Pediatrics, University of California, Los Angeles, Los Angeles, CA, United States

**Keywords:** T cell, cytoskeleton, costimulation, adducin, actin, immune synapse

## Abstract

Alpha-adducin (Add1) is a critical component of the actin-spectrin network in erythrocytes, acting to cap the fast-growing, barbed ends of actin filaments, and recruiting spectrin to these junctions. Add1 is highly expressed in T cells, but its role in T-cell activation has not been examined. Using a conditional knockout model, we show that Add1 is necessary for complete activation of CD4+ T cells in response to low levels of antigen but is dispensable for CD8+ T cell activation and response to infection. Surprisingly, costimulatory signals through CD28 were completely abrogated in the absence of Add1. This study is the first to examine the role of actin-capping in T cells, and it reveals a previously unappreciated role for the actin cytoskeleton in regulating costimulation.

## Introduction

Optimal T cell activation requires the recognition of cognate peptide-MHC by the T cell receptor (TCR) and additional signals from membrane-bound costimulatory receptors such as CD28. Ligation of CD28 by CD80 or CD86 results in recruitment of PI3K to its cytoplasmic tail and activation of the kinase Akt (1). Downstream targets of Akt include the kinase IKK, which activates the NF-κB pathway, leading to IL-2 production in T cells. CD28 is also required for positioning of PKC-θ at the immune synapse (2). PKC-θ contributes to NF-κB activity via phosphorylation of CARMA1 (also known as CARD11) (3). Thus, multiple pathways downstream of CD28 promote NF-κB activation and IL-2 production in recently activated T cells.

T cells rapidly migrate through secondary lymphoid organs, and upon antigen recognition, reorient toward the antigen presenting cell (APC) and form an immune synapse. Both motility and immune synapse formation are actin-dependent processes, making the ability to rapidly reorganize the actin cytoskeleton a critical aspect of T-cell function. A number of cytoskeletal regulatory processes have been studied in T cells, including WASP and Arp2/3-mediated branching and cofilin-mediated severing of F-actin (4). Capping F-actin is another means of regulating the actin cytoskeleton. When actin capping proteins, which include CapZ (also known as capping protein) and the adducin family, bind to the fast-growing barbed end of F-actin, additional actin monomers are prevented from lengthening the filament. This activity grants cells another level of spatial control over actin polymerization.

Alpha-adducin (Add1) differs from CapZ in that it also functions to recruit spectrin to the actin cytoskeleton (5). This activity links the dynamic actin cytoskeleton to the spectrin cytoskeleton which underlies the plasma membrane. Among cells of the hematopoietic lineage, adducin proteins, which exist as heterodimers and tetramers of alpha-adducin with beta- or gamma-adducin, have been shown to be critical components of the cytoskeleton of platelets and red blood cells. In red blood cells, loss of Add1 causes a loss of structural integrity resulting in spherocytosis (6). Although expressed at high levels in lymphocytes, the role of Add1 in T-cell biology has not been examined.

The actin uncapping protein RLTPR (also known as CARMIL2) has been shown to play a crucial role in CD28-mediated T-cell costimulation (7). Interestingly, although RLTPR binds to and negatively regulates CapZ, this activity was recently shown to be dispensable for costimulation (8). Thus, the role of actin-capping in T-cell activation, if any, is not clear. Here, we generated conditional knockout mice lacking Add1 in T cells. We show that CD4+ T cells lacking Add1 develop normally but have a profound defect in CD28-mediated costimulation. This defect reduces T-cell proliferation in response to suboptimal concentrations of antigen and decreases cytokine production. These results introduce a new pathway by which T-cell costimulation is regulated by the cytoskeleton.

## Materials and Methods

### Mice

All mice were maintained in specific pathogen-free facilities and used according to protocols approved by the UCLA Animal Research Committee. C57BL/6 (Strain 000664), CD45.1 (Strain 002014), OT-II TCR transgenic (Strain 004194), and OT-I TCR transgenic mice (Strain 003831) were acquired from Jackson Labs. We acquired C57BL/6N embryonic stem (ES) cells that had been injected with an Add1 knockout first allele containing a neomycin selection cassette surrounded by FRT sites and upstream of LoxP sites surrounding exon 2 of Add1 from the European Conditional Mouse Mutagenesis program, as part of the Knock Out Mouse Project. ES were expanded and implanted into B6 albino mice and the resulting pups were eventually bred to C57BL/6 mice. Progeny were genotyped to determine if the transgene was transmitted and were bred to FLP deleter mice to remove the selection cassette (JAX Strain 009086). Add1 conditional KO mice were then generated by breeding to CD4-Cre (JAX Strain 022071).

### Cell Lines and Reagents

The I-A^b^+ B cell lymphoma line LB27.4 was purchased from the American Type Culture Collection. The following FACS antibodies were used in this study: Fc Block, CD8a BV 421, CD45.2 BV 510, B220 BV 605, CD127 AF 488, CD45.1 PE, KLRG1 PE-Cy7, CD25 AF 647, TNF-α AF 488, and IFN-γ AF 647 (all from Biolegend). Anti-alpha-adducin (ab40760) from Abcam was used for western blots. Anti-PKC-θ (C-18), anti-alpha-adducin (H-100; used for microscopy), and anti-phospho-alpha-adducin (Ser 726) antibodies were from Santa Cruz Biotechnology. CellTrace Violet, CellTrace CFSE, and AF 568-labeled phalloidin were from ThermoFisher. Class I (257-264) and Class II (323-339) ovalbumin peptides were from AnaSpec. GolgiPlug was from BD Biosciences. Anti-CD3 (145-2C11) and anti-CD28 (37.51) were from Bio-X-Cell.

### Western Blots

CD4 T cells were lysed in RIPA buffer containing Halt Protease inhibitor cocktail (Life Technologies). Soluble fractions of lysates were run on SDS–polyacrylamide gels and transferred to PVDF membranes. Membranes were blocked with 5% bovine serum albumin and incubated with the appropriate primary and HRP-conjugated secondary antibodies (GE Healthcare). Western Lightning ECL Pro (PerkinElmer) was used as the chemiluminescent substrate. For the acute stimulation experiment, naïve CD4 T cells were coated with 10 μg/mL anti-CD3 on ice for 10 min, washed and incubated at 37°C for various times in the presence of 50 μg/mL goat anti-hamster crosslinking antibody. Cells were quickly centrifuged and lysed as above in the presence of PhosphataseArrest I (G-Biosciences).

### T-Cell Proliferation Assays

CD4 or CD8 T cells were purified from the spleens of Add1 cKO mice using EasySep negative selection (Stemcell). Cells were loaded with Celltrace 488 or Celltrace Violet and grown on 96 well plates pre-coated with the indicated concentrations of anti-CD3, with or without anti-CD28. Alternatively, OT-II^+^ CD4 T cells were loaded with Celltrace and incubated with LB27.4 B cells loaded with the indicated concentrations of Ova peptide. Cells were assayed by flow cytometry on d3.

### Microscopy

*Imaging T cell-APC conjugates*: Naïve OT-II CD4 T cells were combined with LB 27.4 B cells pre-pulsed with 1 μM Ova peptide and centrifuged for 1 min at 500 × *g*. Cells were allowed to interact for 5 min at 37°C and then plated onto Lab-Tek II 8-well chambers coated with poly-D-lysine. The conjugates were then fixed with 4% paraformaldehyde (PFA) for 30 min, permeabilized with 0.1% Triton X-100 for 5 min, and blocked overnight with 5% normal donkey serum. After staining with the appropriate primary and fluorescently-labeled donkey secondary antibodies (Jackson Immunoresearch), imaging was conducted on a previously described spinning disk confocal instrument (9). Three-dimensional reconstructions of image stacks were performed with Imaris (Oxford Instruments).

*Imaging T cells interacting with stimulatory coverslips*: Lab-Tek II 8-well chambers were precoated with 1 μg/mL anti-CD3 and 5 μg/mL anti-CD28 for 2 h at 37°C. Naïve OT-II CD4 T cells were centrifuged onto the coverslips for 1 min at 50 × *g* and allowed to interact for 10 min at 37°C. The cells were then fixed, permeabilized, and stained as above. We obtained z-stacks by spinning disk confocal microscopy with slices separated by 0.2 μm. For colocalization measurements, we used the GDSC Stack Colocalization plugin in Fiji. The Pearson correlation coefficient between F-actin and Add1 was calculated for single confocal slices at the cell-coverslip interface and 1 μm above the interface. The Moments thresholding method was used with the following parameters: 100 permutations; minimum shift, 9; maximum shift, 16; significance, 0.05.

### Listeria Infections

OT-I^+^ CD8 T cells were purified from the spleens of WT or Add1 KO mice using EasySep negative selection. 10^5^ T cells and 3,000 colony forming units of *Listeria monocytogenes* expressing ovalbumin (a kind gift from Matthew Krummel) (10) were injected *i.v*. into CD45.1^+^ recipient mice. On d8 post-infection, spleens were harvested from recipient mice and red blood cells were lysed. Some splenocytes were immediately surface stained for CD8a, B220, CD45.1, CD45.2, CD127, and KLRG1 and analyzed by flow cytometry to measure accumulation of donor CD45.2+ cells and development of short-lived effector and memory-precursor cells. To measure cytokine production, 10^7^ splenocytes were cultured with 10 μm Ova peptide for 6 h in the presence of GolgiPlug for the final 3 hrs. Cells were surface stained for CD8a, B220, CD45.1, and CD45.2 and then fixed with 2% paraformaldehyde. Cells were permeabilized with 0.5% saponin, stained for TNF-α and IFN-γ, and analyzed by flow cytometry.

### Statistics

Permutation testing was used for all statistical comparisons. This method reduces the potential influence of outliers and relaxed the requirement of knowing the distribution of observations by comparing the value of the test statistic to a reference distribution generated from the data themselves, rather than to a standard distribution. We used the permutationTest2 function of the “resample” package of R to calculate two-sided *p*-values and determine the 95% confidence intervals, performing 50,000–100,000 permutations. All average values in this paper are bootstrapped means, calculated using the “bootstrap” function of the resample package in R. All boxes in figures show the bootstrapped mean and the calculated 95% confidence interval. Confidence intervals are calculated using the “CI.t” function of the resample package.

## Results and Discussion

**Figure 1:**
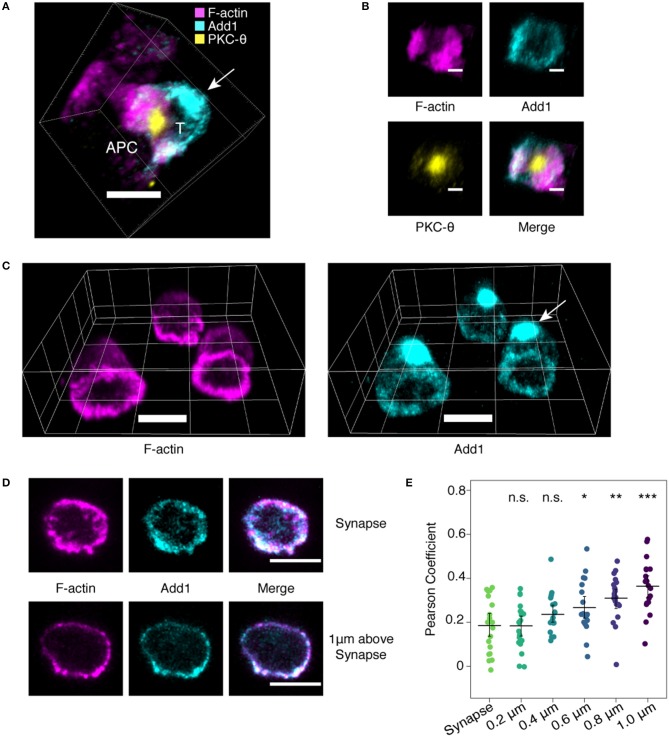
Add1 localizes to the immune synapse and distal pole complex. **(A)** Conjugates formed by naive OT-11 CD4 T cells and peptide-loaded LB 27.4 B cells were stained for Add1, F-actin, and PKC-8. Add1 was found at both the immune synapse and the distal pole complex (arrow). **(B)** Rotated en face view of the T cell-APC interface of the conjugate shown in **(A)**. Add1 and F-actin were located peripherally to PKC-8. **(C–E)** Naive CD4 T cells were allowed to interact with coverslips coated with anti-CD3 and anti-CD28. **(C)** Add1 staining revealed a large accumulation at the distal pole complex (arrow). **(D,E)** Confocal slices showing the distribution of Add1 and F-actin at the T cell-cover- slip interface and 1 μm above the interface. Colocalization analysis revealed a lesser degree of colocalization between Add1 and F-actin at the synapse **(E)**. Pearson Coefficients of Add1 and F-actin colocalization in confocal slices from the synapse to 1 μm above the interface. Add1 colocalization with F-actin progressively increases distal to the synapse. Student's *t*-tests were performed comparing Pearson Coefficients from the synapse to each of the sequential slices. *p < 0.05, **p < 0.01, ***p < 0.001, ns, not significant. Scale bars are 5 μm in **(A,C,D)** and 2 μm in **(B)**.

Given the importance of the actin cytoskeleton in immune synapse formation, we decided to examine the location of Add1 in this context. TCR-transgenic CD4 T cells were incubated with peptide-pulsed APCs and Add1 was stained along with F-actin and PKC-θ. We found that the large accumulation of Add1 at the uropod was reoriented to the distal pole complex opposite the immune synapse (11) (Figure 1A). When viewing the synapse *en face*, from the perspective of the T cell, Add1 was located peripherally, similar to F-actin (Figure 1B). To quantify our observations of the relationship between F-actin and Add1 at the immune synapse, we introduced CD4 T cells to coverslips coated with anti-CD3 and anti-CD28. This method allows for careful quantification of the degree of colocalization since the entire synapse is captured within a single confocal slice. Imaging fixed and stained cells interacting with antibody coated coverslips yielded a similar result to the cell-cell conjugates: a dense accumulation of Add1 at the distal pole complex and rings of both F-actin and Add1 at the interface (Figure 1C). When we measured the degree of colocalization between F-actin and Add1 at the immune synapse and 1 um above the coverslip, we found a significantly greater degree of colocalization distal to the synapse (Figures 1D,E). Together, these results suggest that Add1 serves to restrict actin polymerization at the rear end of migrating cells and at locations distal to the immune synapse in T cells forming conjugates with APCs.

**Figure 2:**
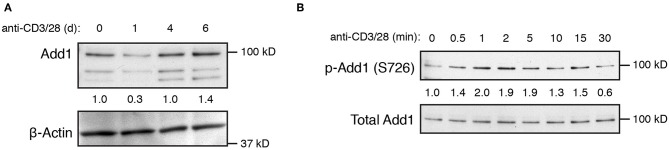
Add1 is rapidly phosphorylated upon TCR stimulation. **(A)** Expression of alpha-Adducin (Add1) was measured in CD4 T cells by western blot *ex vivo* and at d1, d4, and d6 after activation with anti-CD3 and anti-CD28. The two lower molecular weight bands are beta- and gamma-Adducin. **(B)** Phosphorylation of Add1 at Serine 726 was measured in naive CD4 T cells by western blot after anti-CD3 and anti-CD28 stimulation for the indicated times. Results are representative of two experiments.

T-cell activation has been shown to result in a decrease in Add1 expression after 24–72 h of stimulation, indicating that Add1 may play a preferential role in the biology of naïve T cells (12). We also observed a decrease in Add1 levels 24 h after stimulation, however, CD4+ T-cell blasts expressed Add1 at levels similar to naïve T cells (Figure 2A). These results indicate that while Add1 levels vary over the course of an immune response, the protein is present both in the naïve and effector states. Add1 is regulated by phosphorylation at multiple sites. Rho Kinase (ROCK)-mediated phosphorylation at Thr445 and Thr480 enhances binding of Add1 to F-actin (13–15), while F-actin-capping is inhibited by phosphorylation at the C-terminal MARCKS domain by members of the Protein Kinase C (PKC) family (15, 16). Given the importance of PKC-θ activity in T-cell function, we measured phosphorylation of Ser 724 (corresponding to Ser 726 in human Add1) in the MARCKS domain after TCR crosslinking. As seen in Figure 2B, Add1 was rapidly phosphorylated upon T-cell activation. Phosphorylation peaked at 2–5 min and then declined to baseline by 30 min. These results suggest that Add1 plays a role during acute T-cell activation.

To examine the impact of Add1 on T-cell biology, we generated conditional knock-out (cKO) mice using the CD4-Cre system. T cells from these mice showed complete loss of alpha-adducin and also a loss of beta and gamma-adducin (Figure S1A). This effect likely occurs because alpha-adducin pairs with either beta- or gamma-adducin to form stable heterodimers and has been previously noted in global Add1 KO mice (6). Examination of T-cell development in the thymus revealed no defects in single positive, double positive or double negative populations (Figure S1B). Add1 KO mice also had normal percentages of CD4, CD8, CD44+CD62L−, and FoxP3+ Treg cells (Figures S1C,D).

**Figure 3:**
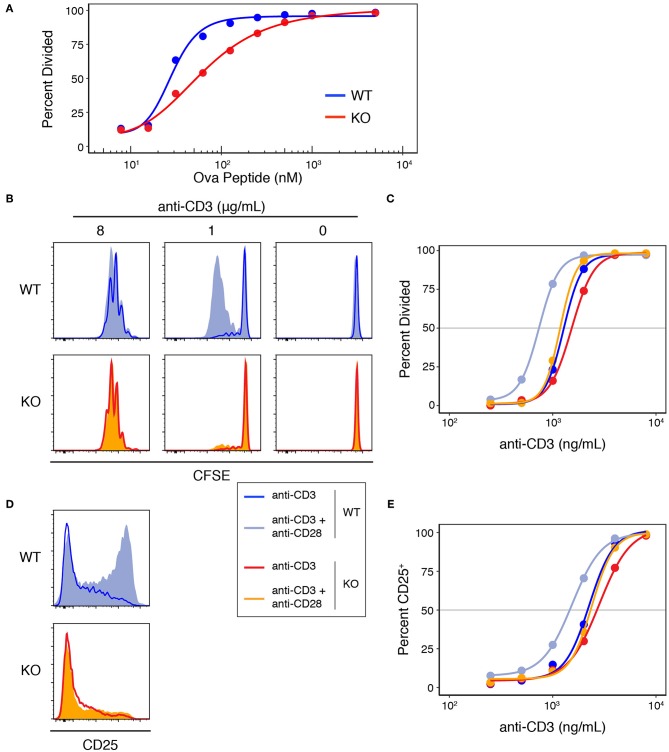
Add1 is required for CD28-mediated costimulation. **(A)** WT and Add1 KO OT-II CD4 T cells were labeled with CFSE and stimulated with LB27.4 B cells loaded with the indicated concentrations of Ova peptide. Cell division was measured on d3. **(B)** CFSE dilution of WT and KO CD4 T cells in response to the indicated concentrations of plate-bound anti-CD3 with or without soluble anti-CD28. Cell division was measured on d3. (C) Percent of cells that have divided at titrated doses of anti-CD3. **(D)** WT and Add1 KO OT-II CD4 T cells were stimulated with 2 μg/mL plate-bound anti-CD3 for 48 h and assayed for CD25 expression. **(E)** Percent of cells positive for CD25 at titrated doses of anti-CD3. Results are representative of >5 **(A–C)** or two experiments **(D,E)**.

To study the role of Add1 in T-cell activation, we crossed Add1 cKO mice to TCR-transgenic OT-II mice that bear a TCR specific for ovalbumin peptide presented by I-A^b^. When stimulated with APCs loaded with titrated doses of peptide, Add1 cKO cells exhibited defective proliferation (Figure 3A). Interestingly, this effect was only seen at intermediate doses of peptide; at maximal TCR stimulation, Add1 KO CD4 T cells proliferated to the same degree as WT. This result suggested either a subtle defect in TCR signaling that only manifested at sub-optimal peptide doses, or a defect in costimulation. To determine if Add1 plays a role in facilitating costimulation, we stimulated WT and KO CD4 T cells with various concentrations of anti-CD3 in the presence or absence of anti-CD28 (Figure 3B). WT and KO T cells proliferated equivalently at low or high concentrations of anti-CD3, regardless of the presence of anti-CD28 (Figure 3C). However, at intermediate levels of TCR stimulation—where costimulation is most critical—Add1 KO T cells were profoundly impaired. Measurement of CD25 upregulation at 48 h post-stimulation showed a similar effect (Figure 3D). Add1 KO T cells showed defective upregulation of CD25 at moderate doses of anti-CD3 and no augmentation when provided anti-CD28 (Figure 3E).

**Figure 4:**
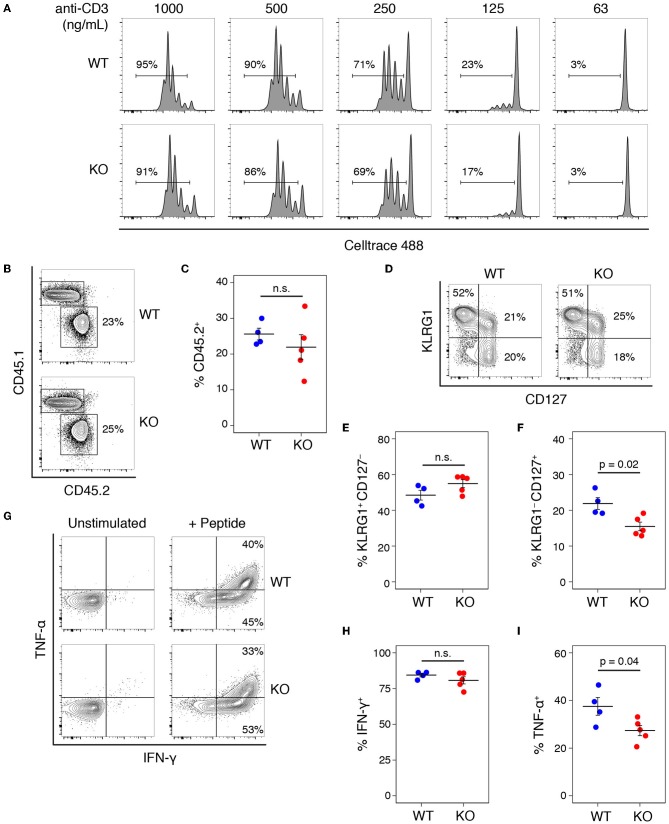
Add1 is not required for CD8 T cell activation or response to Listeria infection. **(A)** CD8 T cells from WT and Add1 cKO mice stimulated with indicated anti-CD3 and 2 μg/mL anti-CD28. **(B,C)** B6 CD45.1 recipient mice were infected with 3,000 CFU of Lm-Ova and 10^5^ OT-1 CD8 T cells from WT or KO mice. On d8, accumulation of donor CD45.2+ cells was measured (B) and quantified for multiple recipients **(C)**. **(D–F)** Among transferred cells, the percentage with a KLRG1+CD127– short-lived effector phenotype and KLRG1–CD127+ memory-precursor phenotype were measured **(D)** and quantified **(E,F)**. **(G–I)** Splenocytes from infected mice were stimulated with Ova peptide and intracellular cytokine staining for IFN-γ and TNF-α was performed. The percentage of IFN-γ + and TNF-α + donor cells was measured by FACS **(G)** and quantified **(H,I)**. Results are representative of two experiments.

We tested whether a similar proliferative defect existed in CD8 T cells by stimulating with a range of anti-CD3 concentrations in the presence of anti-CD28 (Figure 4A), and saw no difference between the proliferative capacity of WT and KO T cells. Comparing the proliferative responses of WT and KO CD8 T cells stimulated with CD3 plus or without CD28 also showed no significant differences (Figure S2). To test the role of Add1 in CD8 cells *in vivo*, we crossed Add1 KO mice with OT-I TCR transgenic mice. We simultaneously infected CD45.1^+^ congenic mice with *Listeria monocytogenes* engineered to express Ova peptide and transferred naïve WT or Add1 KO OT-I cells. Eight days post-infection, we harvested spleens from recipient mice and measured the accumulation and differentiation of donor T cells. We saw no difference in accumulation (Figures 4B,C) or differentiation to KLRG1+CD127− short-lived effectors (Figures 4D,E) and modestly reduced differentiation to KLRG1–CD127+ memory-precursor cells (Figures 4D,F). We also measured cytokine production *ex vivo* and found that similar percentages of WT and KO cells made IFN-γ (Figures 4G,H) and slightly fewer KO cells made TNF-α (Figures 4G,I). These results could be explained by the fact that naïve CD8 T cells are not as dependent on costimulation as CD4 T cells (17). Taken together, it is likely that the role of Add1 in T-cell activation is primarily related to costimulation and not TCR triggering.

To our knowledge, this the first study identifying a role for the actin cytoskeleton in facilitating CD28-mediated costimulation. The actin-uncapping protein RLTPR is critical for costimulation, but only the scaffolding function was required for this role (8). On the other hand, there is well-established role for costimulation in regulating F-actin dynamics at the immune synapse. Blocking CD28 signaling affects the accumulation and centralization of TCR-pMHC clusters and optimal F-actin accumulation at the immune synapse (18). CD28 costimulation appears to impact F-actin dynamics in multiple ways. WAVE2 and HS1, which control actin nucleation, and cofilin, which severs F-actin, showed defective accumulation at the synapse upon costimulation blockade (19). Further, the actin nucleation promoting protein WASp has been shown to promote CD28-mediated F-actin accumulation and CD28 endocytosis (20). Thus, our results indicate that CD28 signaling and F-actin dynamics reciprocally regulate one another.

The mechanism by which Add1 facilitates costimulation will be an important area of future study. It is possible that the actin-capping properties of Add1 are required to correctly orient CD28 at the immune synapse. CD28 containing microclusters form an annular ring around TCR-pMHC accumulations at the immune synapse and recruit PKC-θ (2). We hypothesize that actin-capping is a requirement for this process and experiments with supported lipid bilayers to examine the localization of CD28 and PKC-θ at the immune synapses formed by WT and Add1 KO T cells are ongoing. Given that Add1 links the actin and spectrin cytoskeletons, it is tempting to speculate that reorganization of spectrin plays a role in costimulation. Indeed, antigen stimulation induces membrane-localization of α-spectrin in CD4 T cells (21), and α-II-spectrin has been shown to rapidly reorient to the immune synapse where it colocalizes with F-actin (22). Further experiments will be necessary to dissect the relative importance of Add1-mediated actin-capping and spectrin recruitment in costimulation.

## Data Availability Statement

All data for this study are included in the article/Supplementary Material.

## Ethics Statement

The animal study was reviewed and approved by UCLA Animal Research Committee.

## Author's Note

This manuscript has been released as a Pre-Print at BioRxiv (23).

## Author Contributions

TT and MB: conceptualization, analysis, and writing. TT and HK: investigation and visualization. MB: funding acquisition, project administration, and supervision.

### Conflict of Interest

The authors declare that the research was conducted in the absence of any commercial or financial relationships that could be construed as a potential conflict of interest.
