# Supplementary figures and images for: The Actin-Capping Protein Alpha-Adducin Is Required for T-Cell Costimulation
Source: Front Immunol. 2019 Nov 20;10:2706. doi: 10.3389/fimmu.2019.02706 (PMC6879651; doi:10.3389/fimmu.2019.02706)

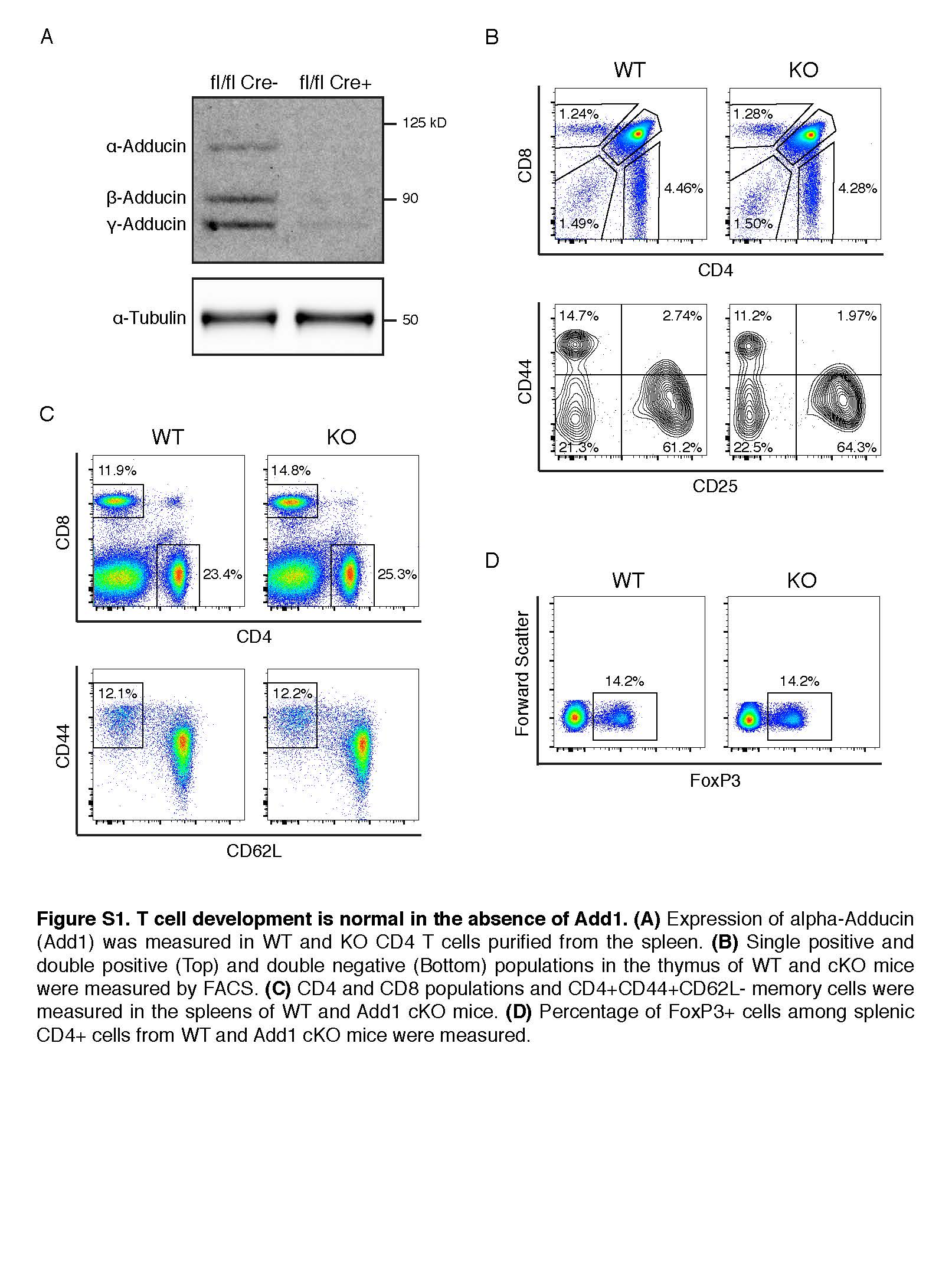

Supplement: Supplementary file 1 [file Image_1.JPEG]

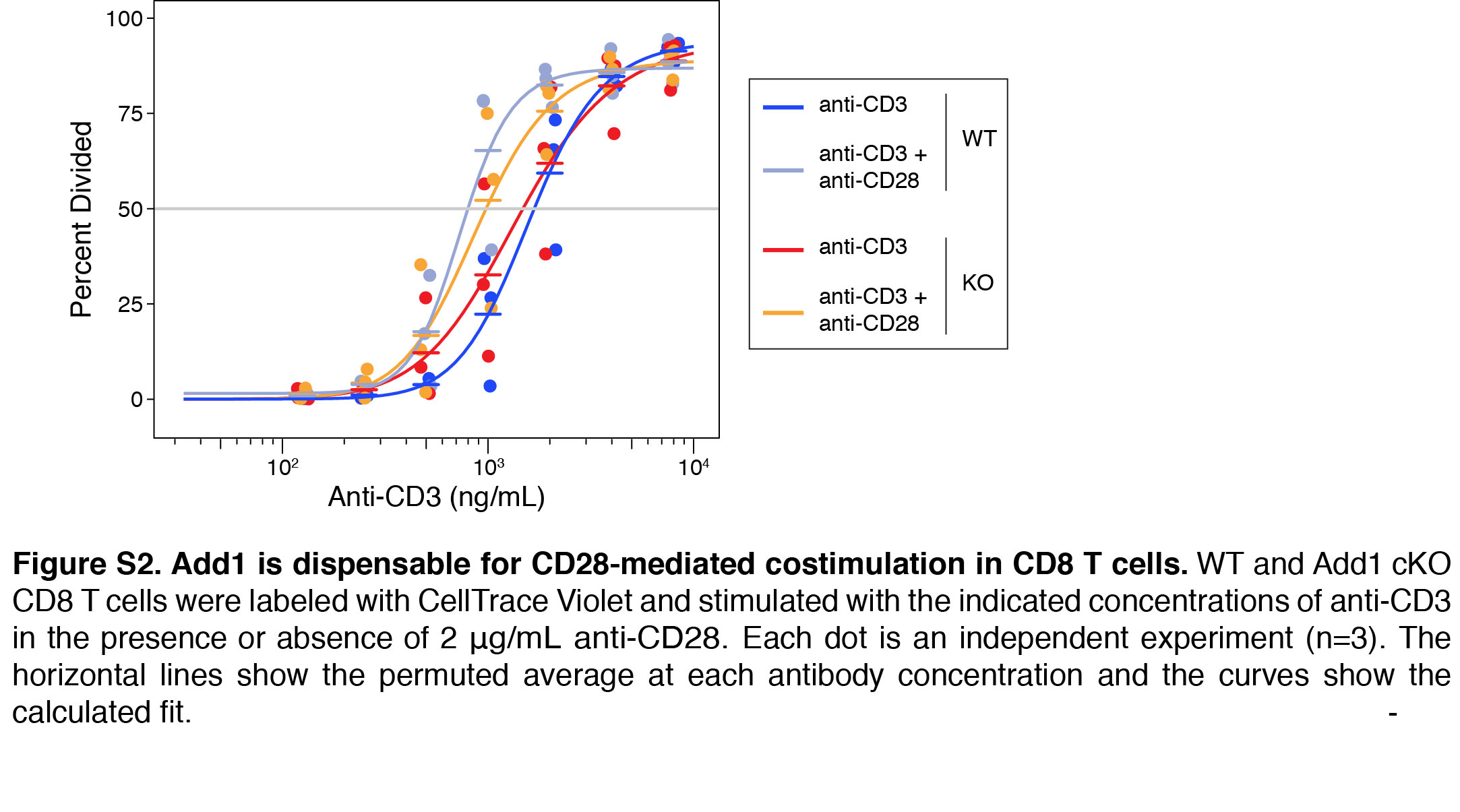

Supplement: Supplementary file 2 [file Image_2.JPEG]
